# Baseline characteristics and progression of neovascular age-related macular degeneration in patients receiving over 60 intravitreal injections of anti-vascular endothelial growth factor

**DOI:** 10.22336/rjo.2023.43

**Published:** 2023

**Authors:** Philipp Prahs, Caroline Brandl, Horst Helbig, Cornelia Volz

**Affiliations:** * Department of Ophthalmology, University Medical Center Regensburg, Regensburg, Germany

**Keywords:** VEGF-inhibitors, age-related macular degeneration, visual acuity, optical coherence tomography, real-life data

## Abstract

**Objective:** Age-related macular degeneration (AMD) is the leading cause of vision loss in older populations of industrialized countries. Antibody-based therapy inhibiting the vascular endothelial growth factor (VEGF) has been very successful in the treatment of the neovascular form of AMD. This retrospective clinical study investigates the baseline characteristics and progression of neovascular age-related macular degeneration (nAMD) in patients who received over 60 anti-VEGF intravitreal injections.

**Methods:** Retrospective analysis of 6812 eyes of 5678 patients undergoing anti-VEGF treatment at our clinic between November 2006 and December 2017 yielded 12 eyes of 12 patients who had received more than 60 intravitreal injections into one eye. We re-evaluated the baseline characteristics of visual acuity, intraocular pressure, optical coherence tomography, fluorescein angiography, as well as autofluorescence and analyzed the documented disease progress as monitored in our daily clinical practice. Data on the fellow eye were also analyzed.

**Results:** Each of our 12 patients had the injected anti-VEGF agent (bevacizumab, ranibizumab, or aflibercept) changed at least once during treatment. After initial improvement, visual acuity decreased in most patients over time. The 2 patients with the best visual acuity at the beginning also showed the best visual acuity at the end of the study. No significant change was observed in the intraocular pressure.

**Conclusions:** After the initial improvement, visual acuity decreased over time. Good visual acuity at the beginning of the study increased the chances of maintaining the same level throughout the treatment. Intravitreal treatment did not affect intraocular pressure.

**Abbreviations: **AMD = age-related macular degeneration, nAMD = neovascular age-related macular degeneration, VEGF = vascular endothelial growth factor, OCT = optical coherence tomography, VA = visual acuity, PDT = photodynamic therapy

## Introduction

Age-related macular degeneration (AMD) is the main cause of irreversible visual impairment in industrialized countries and currently affects about 4 million people in Germany alone [**1**]. In the German State of Bavaria, the prevalence of early AMD in the general population is estimated to be 11.4% and that of late AMD is approximately 0.2% [**2**] in contrast to about 7.2% for late AMD in the age group of 70 and above [**3**]. Late AMD can be differentiated between the dry and the wet form of disease. Intravitreal administration of drugs binding the vascular endothelial growth factor (VEGF) has become the standard therapy for wet neovascular AMD.

Since its first description in 2005 [**4**], intravitreal therapy with different VEGF inhibitors has been administered innumerable times worldwide [**5**]. This treatment slows down disease progression and thus the deterioration of vision for millions of affected patients. However, the impressive success achieved under controlled conditions in the framework of clinical trials could not be replicated in daily clinical practice, and this effect seems to be more pronounced the longer the treatment continues. The frequent visits to the doctor’s surgery are a burden for the patients and their families.

At our clinic, 46525 intravitreal treatments were given between November 2006 and December 2017. This study aimed to identify patients who had received an unusually high number of intravitreal injections and to characterize and compare their baseline findings and disease development to those in scientific literature.

## Methods

Our study included 12 eyes (5 right and 7 left) of 12 patients (7 women and 5 men) aged between 61 and 84 years (median 71.5 years), who had received more than 60 intravitreal injections of VEGF inhibitors because of neovascular AMD, at our clinic, by December 2017 (**Table 1**). 

**Table 1:** Description of characteristics of the study population

	All (n=12)	Men (n=5)	Women (n=7)
Age [years], mean ± SD	72.9 ± 7.6	70,4 ± 29,6	74,7 ± 27,2
Treated eye			
Right % (n)	41.6 (5)	8,3 (1)	33,3 (4)
Left % (n)	58,4 (7)	33,3 (4)	25 (3)
Fellow eye treated % (n)	25 (3)	20 (1)	28,5 (2)
VA [logMAR], median			
Baseline	0,25	0,1	0,3
End of Study	0,7	0,7	0,8
Study time			
Mean study period (months) ± SD	113.25 ± 16.8	115,8 ± 13,7	11,42 ± 19,6
Mean time between injections (months)	1.7 ± 0.3	1,76 ± 0,2	1,65 ± 0,3
The mean number of injections received ± SD	66.25 ± 6.2	64,6 ± 3,7	67,4 ± 7,6

When deciding on a new treatment, our clinic uses a modified pro re nata strategy in agreement with the national guidelines: after a loading dose consisting of 3 intravitreal injections with a VEGF inhibitor, patients are given an appointment for a control visit 4 weeks later. If neovascularization is still active - which is assessed employing optical coherence tomography (OCT) scan or clinical evaluation-, another 1 3 intravitreal injections are recommended. If no sign of activity is observed, patients are given another appointment 4 weeks later and are instructed to ask for an earlier appointment in case of deteriorating vision or increasing metamorphopsia.

Before treatment initiation, every patient underwent a complete ophthalmological examination, fluorescein angiography including autofluorescence measurement, and an OCT scan. Over the years of treatment, the patients were examined at our outpatient department. A full ophthalmological examination of both eyes by an ophthalmologist, as well as an OCT scan were conducted at every follow-up appointment over the years.

OCT scans and fluorescein angiographies were carried out with state-of-the-art commercial equipment from Heidelberg Engineering.

All parameters were retrospectively evaluated from the beginning of the treatment at our clinic until June 2018. Our particular interest was the development of visual acuity and the changes seen in the OCT scans, as well as how intraocular pressure reacted to the frequent treatments.

All data from the intravitreal injections given at the Department of Ophthalmology at the University Medical Center Regensburg were transferred to a database (MariaDB, MariaDB Foundation). Patients were selected for the study using an SQL query. OCT scans and medical letters were identified using the unique patient identification number. Visual acuity and other clinical data (such as ocular pressure) were obtained by manually searching the paper-based patient records. Statistics and graphs were created in Python using the pandas framework.

## Results

*Disease classification and course of treatment*

**Fig. 1:**
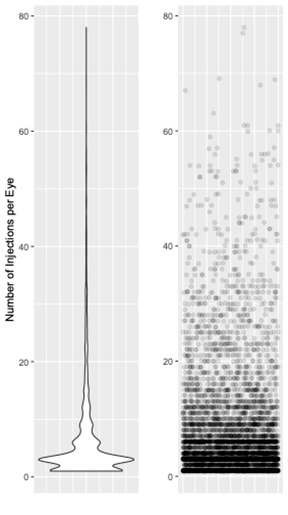
Violin plot (left) and scatter plot (right) illustrating the distribution of intravitreal injections among all patients of the clinic during our study

The mean (+/-SD) number of injections in all the patients who received at least one intravitreal injection in our clinic during the observation period was 6.83+/-8.94. **Fig. 1** illustrates the distribution of the intravitreal injections.

Occult choroidal neovascularization (OCV) was diagnosed in each of the 12 patients included using fluorescein angiography. Treatment duration ranged from 87 to 135 months (mean 113.25 months) (**Table 2**). 

**Table 2:** Detailed clinical parameters of 12 patients receiving over 60 intravitreal injections for the treatment of neovascular age-related macular degeneration

Pat#	IVI no.	BL VA	EOS VA	BL VA fellow eye	EOS VA fellow eye	BL IOP	EOS IOP	Time to reinjection (months)	Study period (months)
1	78	0,2	0,7	1,3	1,3	15	18	1,7	131
2	78	0,1	0,2	1,3	1,3	16	16	1,3	101
3	67	1	0,7	0,2	0,3	16	15	1,5	103
4	68	0	0,3	0,1	0,1	20	17	1,7	121
5	61	0	0,7	1,3	1,3	18	17	2,2	135
6	60	0,3	0,7	0	0,1	15	18	1,7	102
7	62	0,7	0,8	0,4	CF	14	16	1,9	115
8	67	0,3	0,4	0,2	0,2	13	17	1,3	87
9	67	0,1	1	0,8	CF	16	11	1,7	118
10	60	0,2	1	1	0,1	14	16	1,4	89
11	66	0,4	1	1,3	1,3	15	17	1,8	121
12	61	1	1,3	0,5	CF	17	22	2,2	136
logMAR = visual acuity; IVI = intravitreal injection; BL = baseline; VA = visual acuity; EOS = end of study; IOP = intraocular pressure; CF = counting fingers									

**Fig. 2:**
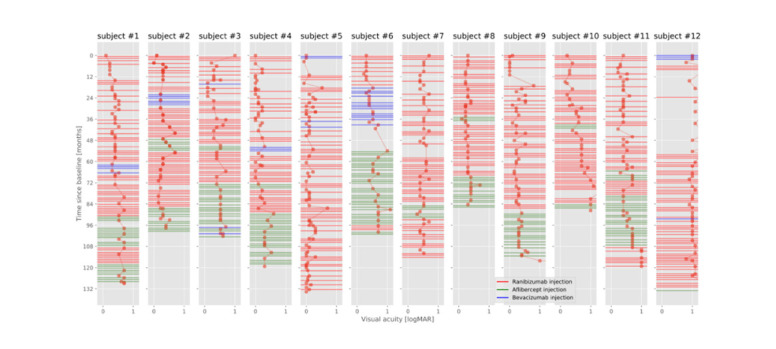
Graphical representation of visual acuity and intravitreal injections. Injections are color-coded as lines corresponding to the different drugs used. Visual acuity values are represented as dots

The administered drug was switched in all patients during that time (**Fig. 2**). 6 patients received each drug available on the market (including bevacizumab for off-label use). In case of suspected tachyphylaxis or failure to achieve the expected results, the administered agent was switched and, after some time, switched back again (**Fig. 2**). At the end of the observation period (December 2017), all included patients were still being treated at our clinic. Over the treatment period, the patients received an intravitreal injection on average every 1.7+/-0.3 months. **Table 1** shows an overview of the most important clinical data for the included patients and **Table 2** provides more data on the course of treatment for each patient.

*Visual acuity*

All patients showed a decrease in visual acuity during the observed period (**Fig. 2**, **Table 2**). Depending on the dynamics of the decrease, 3 groups were distinguished. One group of 5 patients showed a continuous slow decrease in visual acuity from 0.16 (95% CI: 0.08-0.24) logMAR to 0.63 (95% CI: 0.41-0.86) logMAR (patient #1, #2, #3, #6, and #9). Another group of 4 patients (patients #5, #7, #8, and #12) showed long-term stabilization of visual acuity (0.52 logMAR at the beginning [95% CI 0.08-0.97 logMAR] to 0.56 logMAR [95% CI 0.20-0.92 logMAR]). The third group, consisting of 3 patients, showed a rapid decrease in visual acuity either from the beginning (patient #10) or after a stabilization phase (patients #4 and #11). In this group, visual acuity was 0.20 (95% CI 0-0.43) logMAR and 0.94 (95% CI 0.49-1.38) logMAR at the end of the observation period. Visual acuity of patients #4 and #11 had suddenly deteriorated at months 90 and 91. A thorough examination of the paper-based records did not reveal any reason for this rapid decline (such as the development of cataract after lens injury or macular bleeding).

*OCT scans*

**Fig. 3:**
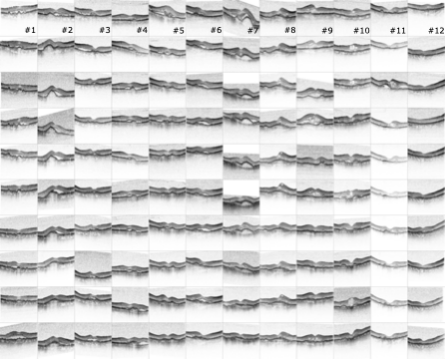
Exemplary OCT image over time showing the development of macular edema or pigment epithelium detachment under therapy with VEGF inhibitors. Patient axis: from left to right, time axis: from top to bottom

**Fig. 3** depicts the results of the OCT scans of the study eyes throughout therapy. The patients had developed both intra- and subretinal fluid accumulations and pigment epithelium detachments. Because these changes fluctuated over time, it was not possible to assign the patients to one of the above-described prognosis groups based on the morphology seen in the OCT scans. At the end of the study, a scar formation was detected in 8 eyes. 

*Intraocular pressure*

Intraocular pressure in the studied eyes was in the normal range, both at the first visit to the clinic (15.7+/-1.9 mmHg) and at the end of our retrospective observation (15.8+/-2.2 mmHg). In the fellow eye, the pressure was also normal (15.7+/-1.8 mmHg at the beginning and 15.3+/-2.2 mmHg at the end).

*Fellow eye*

The fellow eye was also examined on every patient’s visit to the clinic. 4 of the 12 study patients showed changes consistent with dry macular degeneration (drusen and atrophy) and well-preserved visual acuity (0.075+/-0.01 logMAR). 5 patients had a macular scar and poor vision (under 1.3 logMAR). The remaining 3 fellow eyes had also received intravitreal injections for neovascular AMD.

*Adverse events*

There were no cardiovascular or intracranial events in our study population during the observation period. 

## Discussion

Over time, many therapeutic options for AMD have been tested. These included laser photocoagulation, PDT, submacular surgery, radiotherapy, and intravitreal administration of triamcinolone [**6**]. The first therapy to improve vision was the intravenous administration of VEGF inhibitors, which had initially been approved for cancer therapy [**7**]. The breakthrough came when Rosenfeld et al. reported positive results after the first intravitreal application of bevacizumab. The decrease in sub- and intraretinal fluid was documented using OCT [**4**]. Up until present, 3 anti-VEGF agents have been approved: ranibizumab, aflibercept, and brolucizumab. Bevacizumab is still being widely administered as an off-label drug. The efficacy of these drugs has been proven in many clinical studies over time. In a Cochrane review article, Solomon et al. concluded that anti-VEGF therapy is effective in maintaining and improving eyesight [**8**].

Clinical data on the course and outcome of therapy differ from those collected in clinical studies for various reasons. One of them is that patients excluded from clinical studies are still being treated in clinical settings. Another reason is that of the lower number of treatments in clinical practice. Analyzing the information gathered in a routine clinical practice provides important insights into different patient subgroups not previously covered by clinical studies. Such analyses also enable a better understanding of the safety and long-term effects of the therapeutic agents injected.

In this study, we retrospectively analyzed the treatment in our clinic and described our findings without using a standardized protocol or randomization: the control and treatment intervals were different, and the anti-VEGF agent was changed within the observation period of up to 11 years. We aimed to describe patients who had received a high number of injections, similar to that expected in the setting of a clinical study.

*Visual acuity*

In the Seven-Up Study, patients who had taken part in the large clinical studies ANCHOR, MARINA, and HORIZON were examined 7 to 8 years after the start of intensive therapy with ranibizumab. Compared to baseline, 50% of the patients had stable visual acuity, and a third had significantly deteriorated eyesight. 50% of the patients were still regularly treated with intravitreal ranibizumab [**9**].

The SIERRA-AMD study retrospectively examined data from 98,821 eyes of 79,885 patients in the United States, who had received anti-VEGF drugs intravitreally. The improvement in visual acuity achieved in the first treatment year could not be maintained. In the second year, the average visual acuity was below baseline, and patients had lost an average of one letter in each subsequent year [**10**].

A smaller study from Italy including 126 eyes of 109 patients described roughly the same development in visual acuity after a similar number of injections. Visual acuity had dropped below the baseline level in the third year of therapy [**11**]. The number of injections was lower than that of the SIERRA-AMD study (5.2 vs. 9.6 in the first year and 2.3 vs. 6.7. in the third year).

Approximately one-third of the patients included in our study showed a slow decrease in visual acuity, whereas another third had stable values. In all patients, the underlying condition was occult choroidal neovascularization. Gillies et al. described that occult lesions take longer to become inactive than classic lesions, but this fact does not seem to affect the success of the treatment of the level of visual acuity achieved [**12**].

*Intraocular pressure*

Intravitreal injections have been repeatedly reported to not only lead to the expected acute increase in intraocular pressure caused by the injected volume but also to a persistent increase. The most comprehensive meta-analysis including 9,786 patients reported a two-fold increased risk of developing permanently elevated eye pressure [**13**]. In 8.3% of patients, intraocular pressure rose above 21 mmHg and was at least 5 mmHg higher than the previous treatment. Patients with glaucoma had a higher risk of developing increased pressure. The risk seems to increase with the number of injections [**14**]. Possible mechanisms for the pressure increase are drainage disturbances due to microparticles, inflammatory processes, or recurring transient increases in intraocular pressure [**15**]. No relevant increase in intraocular pressure was observed in our patients despite the high number of intravitreal injections given.

*Fellow eye*

A prospective study from 1993 investigated the disease course of the fellow eye in patients with extrafoveal choroidal neovascularization [**16**]. Initially, nAMD was found in more than 50% of fellow eyes and was developed in a further 26% of the fellow eyes over the next 5 years.

Large population-based studies (Blue Mountains Eye Study, Beaver Dam Eye Study, and Rotterdam Study) have shown a rate of 27-68% of nAMD in the fellow eye over 5 years [**17**].

In our study, two-thirds of the patients had bilateral nAMD. Thus, our patient population was at the upper end of the spectrum of the frequency of bilateral disease described in the literature. This finding was probably caused by the longer observation period of up to 11 years. Another third of the fellow eyes in our study showed changes consistent with early dry AMD.

*Geographic atrophy*

VEGF plays an important role in the homeostasis and maintenance of choriocapillaris by the retinal pigment epithelium [**18**]. Thus, therapies blocking VEGF possibly lead to the development and progression of geographic atrophy (GA). When assessing the risk of geographic atrophy in the study population of the CATT-Study [**19**], Grunwald et al. found that poor visual acuity, retinal angiomatous proliferation lesions, foveal intraretinal fluid, as well as monthly dosing and treatment with ranibizumab to be independent baseline risk factors. Grunwald et al. concluded that anti-VEGF therapy may have a role in the development of GA.

Due to the long observation period and the change in the imaging technique over time, we were not able to quantify the progression rate of geographical atrophy in our patients. The qualitative assessment of the documentation indicated scarring (in 8 eyes), but not geographical atrophy, to be the main reason for visual loss.

## Conclusion

Both the results of the clinical studies and the documented treatments confirmed the effectiveness of VEGF-inhibitor therapy. Clinical data depicted the reality of patient care. Observations from long-term use in clinical practice can provide new insights that are unobtainable in clinical studies because of their structure. Such observational data can be used to develop new strategies for increasing patient compliance and thus treatment success. The retrospective analysis of clinical data can contribute to the development of new therapy regimes, as well as improve the planning of further clinical studies. 

In our study, we retrospectively examined data from patients who had required at least 60 intravitreal injections over 7-11 years. We were able to confirm the possible course of disease (improvement, deterioration, and stabilization), as well as of bilateral disease to approximately the same extent as previously described in the literature.

**Conflict of Interest**

The authors declare that they have no conflict of interest.

**Informed Consent and Human and Animal Rights statements**

Due to the retrospective nature of the study, there was no written informed consent required.

**Authorization for the use of human subjects**

Ethical approval: The research related to human use complies with all the relevant national regulations, and institutional policies, is in accordance with the tenets of the Helsinki Declaration and has been approved by the Ethics Committee of the University of Regensburg (25.04.2018: 18-994-104).

**Acknowledgments**

None.

**Sources of Funding**

None.

**Disclosures**

None.
